# A qualitative, cross-cultural investigation into the impact of potentially traumatic work events on Saudi and UK ambulance personnel and how they cope

**DOI:** 10.1186/s12873-022-00666-w

**Published:** 2022-06-27

**Authors:** Khalid Mufleh Alshahrani, Judith Johnson, Lawrence Hill, Tmam Abdulaziz Alghunaim, Raabia Sattar, Daryl B. O’Connor

**Affiliations:** 1 School of Psychology, University of Leeds, Leeds, UK; 2 Psychology department, Faculty of Arts and Humanity, King Abdulaziz university, Jeddah, Saudi Arabia; 3 Bradford Institute for Health Research, Bradford Royal Infirmary, Temple Bank House, Duckworth Lane, Bradford, BD9 6RJ UK; 4 School of Public Health and Community Medicine, University of New South Wales, Sydney, 2052 Australia; 5 School of Health Science, University of East Anglia, Norwich, UK

**Keywords:** PTSD, Potentially traumatic events, Coping strategies, Thematic analysis, Ambulance personnel

## Abstract

**Background:**

Post-traumatic stress disorder (PTSD) is common among ambulance personnel, but its prevalence varies between developed and developing countries. This study aimed to investigate the lived experience of potentially traumatic work events between Saudi and UK ambulance personnel.

**Methods:**

Semi-structured interviews with 16 ambulance workers from Saudi Arabia and the United Kingdom (8 participants from each country) were conducted to explore their lived experiences of potentially traumatic events at work. Data were analyzed using thematic analysis.

**Results:**

Four key themes were identified from interviews: (1) some events are inherently more stressful than others; (2) pressure of organizational and interpersonal stressors; (3) convergence and divergence in cross-cultural coping strategies; and (4) preferring formal and confidential support.

**Conclusions:**

There were differences in the nature of traumatic events and the ways of coping between the two cultures, but paramedics in both cultures had an agreement about their preference for individual and formal support. The results of this study may help inform the development of interventions and PTSD prevention programs for ambulance personnel.

**Supplementary Information:**

The online version contains supplementary material available at 10.1186/s12873-022-00666-w.

## Introduction

Post-traumatic stress disorder (PTSD) is common in first responders who attend accidents and emergencies [1, 2]. PTSD includes 20 symptoms which are organized under four clusters of: (1) intrusion; (2) avoidance; (3) negative cognitions and mood; and (4) arousal [3]. Ambulance workers are one group of first responders, including Paramedics and Emergency Medical Technicians (EMTs), that are the most vulnerable to PTSD [4, 5]. According to reports, PTSD prevalence in these groups ranges from 5.4% in Germany to 94% in Iran [6, 7]. In the last decade, two systematic reviews have estimated that PTSD prevalence in paramedics overall ranges between 11% to 14.6% [5, 8].

However, in their systematic review and meta-regression of PTSD prevalence among first responders internationally, Berger et al. [5] found significant differences in PTSD prevalence between different countries. In particular, they identified that ambulance personnel in developed countries tended to have a lower prevalence of PTSD than those in developing countries. For instance, PTSD rates in ambulance personnel in Italy were 15.7%, in Germany, they were 5.4%, and in the United Kingdom (UK), they were 9.2%. These figures stood in contrast to rates of 89% in Palestinian ambulance personnel, 94% in Iranian ambulance personnel, and 53.6% in Pakistani ambulance personnel [6–12].

In Saudi Arabia, estimates of PTSD prevalence in ambulance workers have varied, but rates are higher than those reported in European countries. One study in Riyadh city estimated PTSD among paramedics from King Khalid Abdul Aziz Medical City (KAMC) and found that 30% of paramedics were experiencing PTSD [13]. Another study among Saudi Red Crescent Authority ambulance personnel who worked in Makkah city found 41% of paramedics were affected by PTSD [14]. The different results between these two studies are probably due to the differences in organizations, locations, and type of self-report questionnaires used. However, despite these elevated rates of PTSD in Saudi ambulance workers, only a small number of studies have investigated trauma experiences in this population. This is concerning, as figures suggest that Saudi ambulance personnel attend to a particularly high number of traumatic events such as car crashes, and as such may be at high risk for developing PTSD [15, 16].

Reasons for the variations observed between countries could include differences in ambulance service organizational structures, differences in questionnaire scale type used, variations in diagnostic classifications, trauma status, sample size and methods [7, 8]. Additionally, lack of public awareness about ambulance work and the provision of work requirements in developing countries could be one of the reasons for the higher prevalence of PTSD in ambulance personnel from these countries [17].

In addition to these organizational factors, it has also been found that psychological and social factors, such as the type of psychological interventions which are available and social support, are associated with PTSD levels in ambulance workers. In their systematic review to estimate the prevalence rate of the mental health problems among ambulance personnel, found that these factors and others can explain the international variation in PTSD prevalence rates in ambulance personnel. However, they have not been adequately studied, and more studies are needed before firm conclusions can be made.

Studies which compare the mental health of paramedics between developed and developing countries are rare. We have identified only one cross-cultural study which has been conducted in paramedics between Saudi Arabia with another country (which was Australia [14]). This study found that rates of depression and PTSD were higher among Saudi paramedics than Australian paramedics [15]. The authors suggested that the higher prevalence of depression and PTSD among Saudi paramedics may be explained by their long working hours, lack of organizational support, lack of appropriate training, and conflict with patients’ family members.

Moreover, there is a lack of qualitative cross-cultural research in ambulance personnel. Qualitative studies which have been conducted in single country samples are informative, suggesting that critical events involving the death of a patient can cause ambulance personnel significant distress but are not events they feel able to disclose and discuss with others [18–20]. A qualitative approach could be particularly beneficial for helping to explain the variation in rates between ambulance personnel in different countries. However, to date, there is no qualitative study that has explored ambulance workers’ mental health between developed and developing countries. Therefore, the current qualitative study aimed to address this gap by conducting a cross-cultural, qualitative investigation exploring the experiences of potentially traumatic work events in Saudi ambulance personnel and UK ambulance personnel. PTSD rates are relatively high amongst Saudi ambulance personnel and lower for UK ambulance personnel. The overall aim of the current study was to investigate the lived experience of potentially traumatic work events between Saudi and UK ambulance personnel. The specific aims were to investigate: 1) the impact of potentially traumatic events on Saudi and UK ambulance personnel; 2) how they coped with these events, and; 3) the types of support they preferred.

## Methods

### Participants and Recruitment

We recruited ambulance personnel, including paramedics and Emergency Medical Technicians (EMTs), who had experienced one or more traumatic events during their work and who had worked for the ambulance service for a year or more. While we confirmed participants’ length of service at the beginning of the interviews, we did not ask participants to confirm that they had experienced a traumatic event. Instead, this was the focus of the interview schedule and so was referred to throughout the interviews. No definition of trauma was provided, so participants each brought their own personal definition of this concept.

We recruited participants from two countries – the UK and Saudi Arabia—who were currently practicing in a clinical capacity via a purposive sampling method. We excluded participants who reported working less than a year because during this period they are usually under practical supervision. Also, volunteers were excluded from participating due to their short working hours. Paramedics responded to study advertisements which were distributed via emailing their organizations and posted on social media (Twitter and Facebook). In the UK, emails were circulated to practice educators by L.H., who has experience as a course director and professional lead of Paramedic Science. In Saudi Arabia, the email was sent to the manager of the Saudi Red Crescent Authority (SRCA) in the Eastern area of Riyadh city.

### Design

This study used qualitative interviews to explore the views and experiences of paramedics in two countries (the UK and Saudi Arabia). A semi-structured interview schedule was developed, containing open-ended questions. The interview schedule consisted of three sections: (1) types of potentially traumatic events experienced at work; (2) strategies used by paramedics to cope with potentially traumatic events and the effectiveness of those strategies; and (3) the type of support or interventions paramedics would like in the future in order to cope with critical situations.

The target sample size of this cross-cultural study was between 12 to 30 paramedics informed by previous studies. According to Guest, Bunce, and Johnson (2006) [21], who conducted 60 interviews in two West African countries, they found that saturation occurred during the first 12 interviews. Similarly, Hagaman and Wutich (2017) [22], interviewed 132 participants in four cultures, and they found that 16 or fewer interviews were sufficient to determine common themes among locations with relatively homogenous communities. Moreover, fewer participants are needed when the sample has more information relevant to the actual study, which is known as “information power” [23].

Semi-structured interviews were chosen to encourage an open dialogue with paramedics when collecting information related to the study aims. All interviews were conducted by K.A. The interview guide was piloted during two mock interviews with J.J. and a professional Saudi paramedic who has more than 15 years of experience. The purpose of the pilot interviews was to improve the fluency of the topic guide. For Saudi participants, all interviews were conducted in Arabic, and transcripts were translated from Arabic to English by the first author (K.A.), an Arabic native speaker from Saudi Arabia. The translation was reviewed by another Arabic native speaker from Saudi Arabia (T.A.).

### Data collection

All interviews were conducted by phone between February to July 2019. Phone interviews were chosen to allow access to paramedics in different locations in each country. Talking about trauma can be a sensitive topic for paramedics who may not want to disclose it in order to avoid stigma and feelings of vulnerability. Nevertheless, interviews provide participants with a chance to talk about their profound personal experiences freely [24, 25]. Therefore, the telephone interviews were considered an appropriate format as they provided a greater level of privacy. Participants have previously reported feeling more secure in phone interviews due to being in their own location rather than restricted by a specific interview location [22, 23]. Participants also report benefit from reduced social pressures and greater anonymity when interviews are conducted via phone [25, 26]. Interviews were translated (for Saudi participants) and transcribed. All translations were conducted by the first author (K.A.) and checked by a second bilingual author for accuracy (T.A.).

### Data analysis

The data was analysed using thematic analysis [27]. This analytical approach is distinguished from other methods by high flexibility in sorting and describing the data set and identifying the most stable themes [27, 28]. Thematic analysis involves six steps. First is familiarisation with the data by reading and rereading all transcripts and writing down the initial ideas. The second step involves generating initial codes; we systematically coded several parts of data, and then the data was organized based on these codes. This phase was completed by K.A. with all transcripts. 30% of transcripts were double-checked and coded by R.S. and J.J. The third step involves developing the themes; in this step, codes were compiled and further developed into higher themes. During the fourth step, potential themes were reviewed during a meeting between the researchers. In this stage, members of the research team (K.A., J.J., R.S.) met and verified that the themes matched to the level of the extracted data. The fifth stage involved defining and naming themes. During this phase, the researchers (K.A. and R.S.) agreed to all final codes by discussion. The final stage involved producing the report to provide a compelling narrative about data based on the analysis. All codes and themes were unpacked, sorted, and organized by using Microsoft Excel 2016 (Bree & Gallagher, 2016).

### Ethics

The study was approved by the School of Psychology Ethics Committee at the University of Leeds, UK (ref no: PSC-578; date approved: January 14, 2019), and the research was performed in accordance with the Declaration of Helsinki for research involving human participants. Paramedics were provided with information sheets and asked to return the consent forms with their signatures to the main author’s email. Participants were informed that they had a right to decide whether or not to take part in the study. Also, they were informed that they had a right to withdraw from the study, including the withdrawal of their data for up to one month after the interview and the right to refuse to answer questions in the interview. Due to the sensitivity of the research topic, paramedics were contacted after the study to ensure that they did not suffer from any psychological harm as a result of their participation. No negative impacts were reported by paramedics as a result of participation in the study.

## Results

A total of 21 potential participants responded to the advertisement, and 16 ambulance workers completed interviews. Three potential participants were ineligible because they were working as volunteers and they had been practicing for under a year. Two withdrew before the interview dates, resulting in a final sample of 16 interviews for analysis: 8 participants from each country. Interviews lasted between 17 and 82 min with a mean of 37 min and 53 secs. All interviews were audio-recorded. The UK participants included three males and five females with a mean age 31.62 (SD = 7.53) from four regions (Northeast, Northwest, Southwest, East of England). While in Saudi Arabia, all interviewees were males with mean age of 32.12 (SD = 3.40) from three regions (southern, western, and the middle region). The range of service years for all participants was between 1.8 to 17.3 years, with a mean 8.05 (SD = 5.20). Thematic analysis identified four main themes and no subthemes (see Table 1).

**Table 1:** Themes, descriptions, and example quotes

*Themes*	*Descriptions*	*Example*
Some events are inherently more stressful than others	There were certain features of events which led them to be more stressful: 1) Events that were unexpected or involving obvious injuries. Both cultures have similar reactions to the same types of trauma. However, the type of event discussed varied between cultures: • In Saudi, participants seemed most impacted by car accidents • In the UK, participants frequently discussed the emotional impact of events including assault, violence, and family disputes 2) Events threatening the safety of ambulance personnel. Both cultures discussed their concern over safety threats, but UK paramedics emphasized this theme more. 3) Events involving vulnerable persons, such as children or the elderly. There were negative emotional impacts on child victims in both countries. Participants in Saudi Arabia reported being affected by events involving female victims	• Traffic accidents are the worst effect on me, and the first accident I faced in my work was the worst one. (SA par 10) • I would say the family events are more stressful, and my worst one would be that a friend of my children got killed on a farm. (UK par 68) • The worst one was when my crewmate and I got attacked by a man with a baseball bat and my crewmate ended up with a fractured skull. (UK par 02) • The hardest event is car accidents, especially if the victims and injured are female because it is difficult to deal with them. They suffer from severe injuries, and at the same time, they do not want to be touch by a strange man. Therefore, I am compassioning with them. (SA par 11)
The pressure of organisational and interpersonal stressors	Organisational and interpersonal stress was described as significant, but the nature and type of this stress varied depending on the culture. Sources of stress in this theme included: • Pressure from colleagues such as bullying or unqualified/inexperienced partners • Organizational pressure such as lack of interest feeling to lose a job or being unprotected from infectious diseases • pressure such as unappreciation and disrespect from people	• I find criticism by colleagues stressful because there is widespread bullying within ambulance services. (UK par 16) • The main management of the authority unfortunately, does not appreciate the work of the paramedic through some policies that apply to the paramedic. (SA par 44)
Convergence and divergence in cross-cultural coping strategies	This theme described the similarities and differences in coping strategies and distraction techniques across cultures * (1) Cross-culturally used techniques:* • Sport activities • Separating between work and private lives • Learning from other first responders * (2)Culturally variable coping techniques:* • Prayer and believing • Gambling • Drinking alcohol with family members or friends	• I run quite a lot, so I find that's a really good way of sorting things out in my head. (UK par 02) • when I'm out of work, I close my phone and do not share my activities with colleagues to forget all the daily events I've been through. (SA par 05) • I knew this strategy from a colleague who works police officer, and as you know, he faces similar cases as I do, and told me that if you have any emotional problems at work do not keep that issue inside your mind, you must speak other about what you faced because you will psychologically suffer. (SA par 11) • Yes, the praying, I use it a lot because when I am uncomfortable with something wrong in my work, or in my life, I prepare to pray. I feel a wonderful comfort even if nothing happens in the subject matter that made me worry because I believe that my God, will guide me to solve this problem. (SA par 14) • I typically find gambling is a good distraction technique as well, so I would sometimes go and just put bets on and just relax for an hour or so. (UK par 14) • I find after the event I will typically try and distract myself. So, there's been times where I've asked family members to go for drinks out just to distract myself, get into a different environment other than the normal home that I come back to. (UK par 14)
Preferring formal and confidential support	This theme explains the preferences of paramedics regarding the support they would like to help them cope with potentially traumatic work events. Their preferences were for:’ • Formal support or intervention • Individual support	• The official intervention feels me that I am under the attention of Red Crescent as a staff member in all psychological and educational aspects. This thing gives me more motivation in my work performance and in everything around me. (SA par 05) • Probably individual treatment I would choose. I think I just feel more comfortable speaking to just one person who would probably understand. I think I'd be more open with him rather than in a big group. I think I'd struggle to speak freely in a big group. (UK par 56)

### Thematic analysis of the interviews

#### Theme one: Some events are inherently more stressful than others

In both countries, certain types of events were described by paramedics as being inherently more stressful to deal with. For example, paramedics in both cultures had similar reactions to events involving exposure to severe physical injuries. All paramedics found traumatic accidents such as car accidents stressful, but these were more frequently reported by Saudi paramedics:

*Traffic accidents are the worst effect on me, and the first accident I faced in my work was the worst one. We received a call about the incident with multiple physical injuries; when arrived at the incident location, we found all victims had died (nine female teachers and the tenth was the driver), except a little girl about three years is only one still alive. (SA par 10)*

All paramedics found incidents involving assault, violence, and family conflict stressful. They also found incidents and accidents within family particularly stressful, but all these types of events were more frequently encountered by UK paramedics:

*My worst one would be that a friend of my children got killed on a farm. So, it was a three-year-old, but it was a child that I knew basically got run over by a tractor, which his dad was driving. (UK par 68)*

Paramedics described events that presented a threat to their own physical safety as particularly stressful, although these were more frequently discussed by UK paramedics than Saudi paramedics:

*I would have said probably the most stressful event would be assaults, like being assaulted on the job. Yeah, probably the worst one was when my crewmate and I got attacked by a man with a baseball bat, and my colleague ended up with a fractured skull. (UK par 02)*

Events involving vulnerable victims such as children and elderly people were reported as stressful by paramedics in both cultures. However, Saudi participants also discussed being impacted by events including female victims. This appeared to be due to religious and cultural reasons, and the fact that all Saudi paramedics are men:

*The hardest event is car accidents, especially if the victims and injured are female because it is difficult to deal with them. They suffer from severe injuries, and at the same time, they do not want to be touched by a strange man. Therefore, I am compassionate with them. (SA par 11)*

#### Theme two: The pressure of organizational and interpersonal stressors

Cross-cultural, organizational, and interpersonal pressures were significant factors influencing how well paramedics coped with the traumatic events they faced when working. The nature and severity of these pressures varied between the UK and Saudi participants. Three main pressures were recorded. The first of these was pressures from colleagues/co-workers including bullying, unqualified partners, and partners who lacked experience. These stressors were more frequently reported by UK paramedics:

I find criticism by colleagues stressful because there is widespread bullying within ambulance services. (UK par 16)

However, Saudi paramedics also reported experiencing stress related to their co-workers, such as irritable paramedics.

*I worry when I work with some colleagues who quickly get angry, especially when the relatives of the patients get out of control because my focus then is divided on doing my [own] work, observing [my colleague’s] work, and trying to calm [my colleague] down. (SA par 88)*

The second was pressure from the organization including ambulance personnel feeling afraid of losing their job due to strict organizational rules. This pressure was described by paramedics in both cultures but was more commonly discussed by Saudi participants who suffered from unfair organizational policies (e.g., the paramedics are responsible for any damage of ambulance cars). This included not insuring ambulances against traffic accidents and asking the paramedics to repair them since they were responsible for the ambulances. In addition, it also included not providing a financial incentive which is usually added to their salaries as standard (known as a vulnerability-to infection allowance):

*The main management of the authority, unfortunately, does not appreciate the work of the paramedic through some policies that apply to the paramedic. (SA par 44)*

The third factor was pressure from society. Paramedics from both countries described feeling underappreciated by the public and sometimes experiencing members of the public interfering or interrupting them when they were undertaking their duties in public places. This pressure was more frequently described by Saudi participants, who felt that an increase in appreciation would have reduced the impact of stressors on them:

*If most people appreciate and realize the difficulty of the ambulance work and not everyone can do this work, this in itself is a great support that gives me greater motivation to do the best. (SA par 11)*

However, this pressure was also referred to by some UK paramedics, who said working while there are many people around the incident site put them under heightened pressure because all eyes are on the paramedic:

*Working in front of a load of people there puts you under a lot of pressure, which means that you feel like you’re unable to make as many mistakes compared to a different job, it’s more stressful, and I think it’s more emotional stress. (UK par 02)*

#### Theme three: Convergence and divergence in cross-cultural coping strategies

This theme captured the similarities and differences in coping strategies used by paramedics in both cultures. Some strategies were used by paramedics in both countries. One of these included sports activities, which were used as a distraction technique in both cultures, with different types of sports preferred by paramedics in each culture. Participants from the UK used running and swimming, while Saudi participants walked and went to the gym after finishing their shifts:

I run quite a lot, so I find that’s a really good way of sorting things out in my head. (UK par 02)

*I find walking is the best way to adapt quickly to work issues. So, when I think of a stressful accident after work, I go home and change my clothes and walk alone on the Corniche (a place overlooking the sea) for a long-distance sometimes up to 4 km. (SA par 88)*

In both countries, paramedics also described consciously separating their professional and personal lives in order to avoid work stressors when they are not working:

When I’m out of work, I close my phone and do not share my activities with colleagues to forget all the daily events I’ve been through. (SA par 05)

*I’m not talking about the more serious jobs I go to my wife because I don’t want to bother her. I don’t want her to deal with the things I have to deal with. So, I try not to bring business events with me into my home or even into my private life. (UK par 17)*

Participants in both countries reported learning how to cope with these events from their relatives and friends who worked in similar jobs as first responders by observing or seeking advice:

*I knew this strategy from a colleague who works as a police officer, and as you know, he faces similar cases as I do; and told me that if you have any emotional problems at work, do not keep that issue inside your mind, you must speak other about what you faced because you will psychologically suffer. (SA par 11)*

*I don’t talk to my mum about it particularly. I might talk to my dad. My dad was a police officer, and he so has had some similar experiences. He gave me some good advice to do the best, but he retired nearly 20 years ago. So, his memories are from a long time ago, really. (UK par 17)*

In contrast, there were three coping strategies that were only reported by paramedics in one of the two cultures. One of these was prayer and spirituality, which was only described by Saudi paramedics. Saudi Arabia is a Muslim country, and as such, all participants who described using this coping strategy referred to praying and reading the Quran (the Muslim holy book) and found this a helpful and beneficial practice:

*Yes, the praying, I use it a lot because when I am am uncomfortable with something wrong in my work, or in my life, I prepare to pray. I feel a wonderful comfort even if nothing happens in the subject matter that made me worry because I believe that my God will guide me to solve this problem. (SA par 14)*

The remaining two strategies could be regarded as potentially risky or harmful and were only reported by UK paramedics. The first of these was gambling; the participants who used this described finding it to be a useful distraction that helped them to take their minds off the stressful work events they had experienced:

*I typically find gambling is a good distraction technique as well, so I would sometimes go and just put bets on and just relax for an hour or so. (UK par 14)*

UK paramedics also described drinking alcohol to help them cope with potentially traumatic work events. The participants who reported using this strategy said they did this in conjunction with socialising with family or friends and found that it provided a useful distraction from unhelpful thoughts of work:

*I find after the event; I will typically try and distract myself. So, there’s been times where I’ve asked family members to go for drinks out just to distract myself and get into a different environment other than the normal home that I come back to. (UK par 14)*

#### Theme four: Preferring formal and confidential support

This theme describes the preferences which paramedics had for the kind of support they would prefer to be offered after being involved in a potentially traumatic work event. Interestingly, preferences were similar in paramedics from both countries, with both groups expressing a preference for formal support and interventions provided by their organization over informal, ad-hoc support from colleagues or supervisors. Paramedics felt that the offer of formal support would increase their sense of being valued by their organizations:

*An official intervention makes me feel that I am under the attention of my organization as a staff member in all psychological and educational aspects. This thing gives me more motivation in my work performance and in everything around me. (SA par 05)*

*I like both, but the formal support was what actually really helped me, in the end, is having a stranger who knows what they're doing, in terms of psychological support, to help. That’s what sort of actually really sorted me out in the end. (UK par 68)*

Individual support was also preferred by both UK and Saudi participants rather than group support in order to protect their privacy:

*Probably individual treatment I would choose. I think I just feel more comfortable speaking to just one person who would probably understand. I think I’d be more open with him rather than in a big group. I think I’d struggle to speak freely in a big group. (UK par 56)*

It is better individually because I will be more comfortable talking in case it is talking to someone else and protecting the privacy. (SA par 27)

## Discussion

The main aim of this study was to investigate the lived experience of potentially traumatic work events between Saudi and UK ambulance personnel, in order to identify: (1) the impact of these potentially traumatic events on ambulance personnel in both cultures; (2) to understand how they cope with these events; and (3) to gain insight into which type of support they preferred. The results found four key themes, which suggested that: (1) some work events were inherently more stressful than others, particularly those involving physical injuries, vulnerable victims or threats to the paramedics themselves; (2) organizational and interpersonal stressors such as incivility compounded the impact of stressful events; (3) there was both convergence and divergence in coping strategies between cultures, with physical activity used by paramedics in both countries, but spiritual coping only used by Saudi paramedics; and (4) all paramedics preferred formal and confidential support to informal support or group interventions.

Previous studies have used qualitative methods to examine how ambulance personnel experience and cope with potentially traumatic work events, but none has compared experiences of these events between ambulance personnel in two different cultures. We found that paramedics reacted to comparable types of events in similar ways, but exposure to event types varied. Saudi ambulance workers frequently discussed the stress they experienced in response to car accidents, and this may be because car accidents are common in Saudi Arabia, with one person dying and four injured every hour in a car accident [29]. According to the World Health Organization [30], Saudi Arabia reported the highest rate of road deaths and injuries of all high-income countries in 2016, with an estimated 28.8 per 100,000 population, compared with a world average of 17.7 per 100,000 population. Moreover, traffic accidents were the most common cases that were treated by Saudi paramedics based on the report of the Saudi Red Crescent Authority in 2019 [31]. In contrast, UK ambulance personnel instead recounted potentially traumatic work events involving victims of assault, violence, and family disputes. This is consistent with a study by Alexander and Klein (2001) [32], which investigated the prevalence of psychopathology among the UK personnel workers and its relationship to their exposure to critical incidents. The study found that the most stressful and disturbing incidents for ambulance personnel were those involving child victims, known victims to the ambulance crew, and severe injuries. Both the UK and Saudi participants also described concerns about events that threatened their safety. These concerns emerged more strongly among ambulance workers from the UK and were consistent with the results of two previous systemic reviews [33, 34] that found an increasing prevalence of violence against EMTs and injuries at work between them.

However, it is possible that these types of events are less frequent for Saudi paramedics due to strict penalties imposed by the Saudi Ministry of Health against attacking and assaulting all health practitioners, including the ambulance personnel. These legal penalties may lead to ten years in prison or paying one million Saudi riyals (approximately £200,000) [35]. Furthermore, the ambulance personnel in both cultures were negatively impacted by events with vulnerable victims, which is consistent with previous studies [18, 32, 36–39]. These findings expand previous knowledge by showing that similar types of events have comparable impacts on paramedics cross-culturally; however, exposure to different event types varies.

The findings of the current study also showed that there are some cross-cultural differences in the sources of organizational and personal stress among ambulance personnel. In the UK, the ambulance workers were more impacted by pressures from their colleagues (e.g., bullying, blaming, or not performing their duties properly), which is in line with some previous studies. For example, bullying of NHS ambulance workers in the UK has been found to be the result of strict administrative practices, increased work demands, and reduced resources (e.g., [40, 41]). While Saudi ambulance workers suffer less from such pressures, they reported feeling more organizational pressures than their peers in the UK. This finding contrasts with the results from a recent qualitative study [42] which found that Saudi EMSs were satisfied and happy with their job in SRCA. This inconsistency may be due to the fact that the study by Alanazy, Fraser, and Wark (2021) [42] focused on the comparison between the aspects of financial and administrative support among EMSs in rural and urban areas, while our study is concerned with psychological support, in particular, creating and developing intervention and prevention programs based on coping strategies and support preferences used by paramedics.

In terms of coping strategies, the current study found that Saudi ambulance personnel used religious coping mechanisms, whereas UK ambulance workers did not. This finding is consistent with a previous study by Koenig et al (2014) [43], which found that prayer and reading the Holy Qur’an are popular strategies to treat and diminish stressful events among Saudi people. The UK ambulance personnel used different distractions such as gambling and drinking with others. It is also consistent with studies showing that gambling and drinking have been used as distractions among paramedics and EMSs in the UK to cope with stressful events [44–49]. However, these findings extend existing research by showing that culture is a powerful influencing factor in leading people to identify appropriate methods to cope with stressful events. They also suggest that psychological interventions designed to support ambulance workers will need to be sensitive to these cultural variations. Future interventions might consider incorporating spirituality for Saudi paramedics while recognizing and discussing the potential for risky or harmful coping strategies in UK paramedics.

Ambulance personnel in both cultures preferred formal organizational support, despite most Saudi participants indicating that they did not receive any formal organizational support [50]. This aligns with previous research, which has suggested that after critical events, most paramedics and EMTs prefer workplace interventions [51–53], and this may help them to cope with the emotional impact and regain control and confidence in their performance [54]. Also, the current study found that the ambulance personnel in both countries preferred individual interventions rather than group interventions such as [55]. This adds to the existing knowledge base on intervention preferences for ambulance personnel and may be due to a desire to protect their privacy and avoid stigma from others [56],

## Strength and limitations

This is the first qualitative study to compare the experiences of ambulance personnel in these two different cultures: the UK as a developed country and Saudi Arabia as a developing country. It benefited from the use of a diverse research team including both Saudi and UK natives and multiple bilingual speakers to check data and translations. However, findings may be limited due to self-selection bias as all interviewees volunteered to participate and may not reflect the majority of ambulance workers in each country. It is also limited by the use of semi-structured interviews to collect data. It is possible that an observational approach may have enriched the findings, but due to the nature of ambulance work, this was not possible in either country.

## Conclusion

Few studies have investigated the potentially traumatic events among ambulance personnel and how they cope during and after these events have occurred. This cross-cultural study compared ambulance personnel in the UK and Saudi Arabia to try to understand paramedics’ views about which traumatic incidents they experience, coping strategies they use, and the support they prefer to deal with these events. There were differences in the nature of traumatic events and ways of coping between the two cultures, but paramedics in both cultures had agreement about their preferences for individual and formal support. The results of this study may be used by organizations that are responsible for ambulance services to improve the performance of ambulance workers by monitoring potential traumatic events and designing prevention and intervention programs to deal with them.

## Supplementary Information

**Additional file1:** **Appendix 1.** The interview schedule.

## Data Availability

Due to the in-depth, personal nature of the interviews, whilst all transcripts were anonymised, it is possible that individuals could be identified from interviews by others who are familiar with them. As such, to protect participants’ confidentiality, we are not making the data available. For further information, contact ml17kma@leeds.ac.uk.
