# Supplementary material for: A qualitative, cross-cultural investigation into the impact of potentially traumatic work events on Saudi and UK ambulance personnel and how they cope
Source: BMC Emerg Med. 2022 Jun 27;22:116. doi: 10.1186/s12873-022-00666-w (PMC9235175; doi:10.1186/s12873-022-00666-w)
Supplement: Supplementary file 1 — Additional file1: Appendix 1. The interview schedule. [file 12873_2022_666_MOESM1_ESM.pdf]

Appendix 1. The interview schedule

Opening

Hello, my name is Khalid. I'm a researcher from the University of Leeds. This is the time we agreed to speak by email – is this still ok with you?

Before beginning the interview, I would like to give you some information about this research. I am interested in understanding the stressful events paramedics experience, how you cope with these and the support you would like. In this interview, I will ask you some questions about these topics. It is important to know that this interview is only for research purposes and if there anything you would rather not respond to, you can just decline to answer.

Timeframe: This interview should take approximately 45 minutes to complete.

Have you had a chance to read the Participant Information Sheet?

[Check participants are happy with the information on the Participant Information Sheet and take informed consent, which will be recorded]

To begin with, can you tell me a little bit about yourself? ***[Demographics: the demographics will NOT be recorded]***

- What is your gender?

- What is your age?

- How many years and months have you been working in ambulance services?

- How would you describe your ethnicity?

- Which region do you work in?

- How many hours do you work on average in each week?

SECTION 1: Types of stressful events experienced at work *(this sections aims to explore the nature of stressful events in paramedics' work and the features of these events which paramedics find most stressful)*

-Which kinds of events do you find stressful at work?

-Can you describe any particular stressful events you've experienced?

- What was it about this/these events that you found difficult?

-What thoughts did you have about this/these event/s afterwards?

- In which other ways did this/these event/s affect you? (Emotional or physical effects)

-How long did you have these thoughts/emotions/other problems after the event/s?

- How did this/these event/s affect your performance in work?

- How did this/these event/s affect your personal life?

  -How did they affect your family life?

  -How did they affect your social life?

SECTION 2: Coping strategies *(This section aims to uncover the ways in which paramedics cope with stressful event and how effective these coping strategies are)*

1. Which things helped you to cope with the event when it was happening?

  -Do you have any coping strategies you use?

  - How did you learn this/these coping strategy/strategies? (education? experience?)

  -How helpful did you find using this/these strategy/strategies?

2. Which things helped you to recover from the impact of this/these event/s?

  -Did you organisation provide you with any formal support like a debriefing meeting or one-to-one psychological support?

  -Did you receive any support from colleagues?

  -Did you receive any support from family?

  -Did you use any self-help or seek psychological support yourself?

  -How helpful did you find this/these support/strategies? Would you do the same thing again in the future?

SECTION 3: Preferences for support *(this section aims to uncover the types of support or interventions paramedics would like to help them cope with critical events In the future)*

[For paramedics who received formal support from their organisation]

1. Which things did you like about the Support/intervention your organisation provided you with?

2. Would you want to be offered something similar in future?

3. Would you rather be offered something different in future? How would it be different?

[For paramedics who received no formal support from their organisation]

1. What kind of support or help would you have liked your organisation to offer you?

-Would you have appreciated a formal intervention or rather better informal support (e.g., from colleagues)?

-Who would you want to provide this (a supervisor? A senior paramedic from another organisation? A psychologist?)

- If you would want a formal intervention, what format would you want to receive this intervention in? (Individual or group?)

-If you would want a formal intervention, what kind of things would you want the intervention to include (e.g., psychological training, or therapeutic support)?

- If you would prefer more informal support, how do you think this could be improved?

Closing

I appreciate the time you took out to take part in this interview. Is there anything else you would like to add before we finish this interview?
Thank you again, your responses will be very helpful.
